# RBApy: extending resource allocation modeling to eukaryotes in complex environments

**DOI:** 10.1093/bioadv/vbag276

**Published:** 2026-09-17

**Authors:** Oliver Bodeit, Nadia Bessoltane, Delphine Charif, Anaghim Temtem, Olivier Inizan, Anne Goelzer

**Affiliations:** Université Paris-Saclay, INRAE, MaIAGE, Jouy-en-Josas, France; Université Paris-Saclay, INRAE, AgroParisTech, Institute Jean-Pierre Bourgin for Plant Sciences, Versailles, France; Université Paris-Saclay, INRAE, AgroParisTech, Institute Jean-Pierre Bourgin for Plant Sciences, Versailles, France; Université Paris-Saclay, INRAE, MaIAGE, Jouy-en-Josas, France; Université Paris-Saclay, INRAE, MaIAGE, Jouy-en-Josas, France; Université Paris-Saclay, INRAE, MaIAGE, Jouy-en-Josas, France; Univ Toulouse, INRAE, MIAT, Castanet-Tolosan, France

## Abstract

**Motivation:**

Resource allocation modeling—as the Resource Balance Analysis (RBA) framework—provides a way to understand and predict how limited cellular resources (e.g. energy, proteins, etc.) in cells are distributed among competing cell processes within a limited cellular space. Currently, resource allocation modeling for eukaryotes remains limited due to the lack of software capable of generating calibrated RBA models for these types of cells, unlike prokaryotes, which benefit from the software tools such as RBApy, RBAtools, and the RBAml format for model encoding.

**Results:**

Here, we extended the RBA toolkit (RBApy, RBAtools, and RBAml) to account for specific aspects of eukaryotic cells growing in complex environments such as varying temperature, light, or nutritional conditions. We used them to generate and simulate RBA models of both prokaryotic (*Escherichia coli*) and eukaryotic (*Arabidopsis thaliana*) cells for varying temperatures. The resulting models show excellent prediction capabilities when benchmarked against published experimental datasets. The upgraded RBA toolkit will pave the way to creating, calibrating, and running resource allocation models for crops, livestock, or humans for a wide range of medical, biotechnological, or agricultural applications in the future.

**Availability and implementation:**

RBApy and RBAtools are available via PyPI and at https://github.com/RBAgroup.

## Introduction

Eukaryotic cells such as those of plants, animals, and fungi are complex systems with multiple organelles, intricate metabolic pathways, and complex regulatory networks. Resource allocation models provide a way to understand and predict how limited resources (e.g. energy, nutrients, proteins, etc.) are distributed among competing cell processes, such as growth, maintenance, stress response, and reproduction, within a limited cellular space. Among different existing resource allocation modeling frameworks (Dourado *et al*. 2026), Resource balance analysis (RBA; Goelzer *et al*. 2011, Goelzer and Fromion 2019) formalizes the resource allocation between competing cell processes as a linear convex optimization problem (Nesterov and Nemirovskii 1994), and predicts for a specific environment the set of feasible cellular phenotypes compatible with the available extracellular resources. Parsimonious resource allocation between competing cell processes is modeled mathematically by maximizing cell growth, thus obtaining the optimal growing cell phenotype. Under this assumption, RBA has provided accurate predictions of cell phenotypes for yeast (*Saccharomyces cerevisiae*; Dinh and Maranas 2023) and plant leaf cell (*Arabidopsis thaliana*; Goelzer *et al*. 2024). Two software tools, RBApy (Bulović *et al.* 2019) and RBAtools (Bodeit *et al*. 2023) were previously developed and used for generating, calibrating and simulating RBA models for prokaryotic cells such as *Bacillus subtilis* (Goelzer *et al*. 2015), *Escherichia coli* (Bulović *et al.* 2019), *Vibrio natriegens* (Coppens *et al*. 2023), or *Cupriavidus necator* (Jahn *et al*. 2021). RBApy takes an annotated genome-scale metabolic model (GEM; Thiele and Palsson 2010) as its input and automatically enriches the metabolic network with cell processes such as translation or protein folding, enabling rapid and semi-automated model generation. The models can be further refined systematically (using curation files) and calibrated if user-supplied datasets (such as quantitative proteomics) are provided. RBAtools provides a versatile programming interface for RBApy, enabling the implementation of custom simulation workflows.

Nevertheless, RBApy (version lower than 3.0) currently lacks functionality to handle some specific challenging properties of eukaryotic cells, such as the subcellular compartmentalization into organelles, the multiple localization of molecules (including metabolites and proteins) and cellular processes inside these organelles, the recycling of resources during stationary phase, or variable organelle sizes, to cite a few. Here we upgraded RBApy (version 3.0) and RBAtools (version 2.0) to include such aspects of eukaryotic cells in RBA models. To ensure backward compatibility, we extended the XML-based file format (RBAml) for the encoding of RBA models to support the new features, and upgraded all existing RBA models of prokaryotic cells (available at rba.inrae.fr). Finally, to demonstrate RBApy’s new capabilities, we generated RBA models of *Arabidopsis thaliana* and *Escherichia coli* accounting for temperature dependence and used them to simulate their adaptation to varying temperatures.

## What is new in RBApy?

### Model construction

Cells, and especially eukaryotic cells, need to grow and survive despite a wide range of abiotic stresses (e.g. nutritional, thermal, or saline stress). To model this, we extended RBApy with functionalities to account for (i) the impact of abiotic stresses and (ii) the specific physiology of eukaryotic cells, especially under conditions in which cells do not grow, i.e. during the stationary phase.

#### Abiotic stress

Environmental variables such as temperature, pH, or light can now be defined along with the nutritional composition of the cell environment. As in Bulović *et al.* 2019, the impact of stress is mainly modeled by (i) stress-related cellular processes within the model (e.g. proteolysis, protein folding, stress detoxification) and by changes in the catalytic rates of molecular machines such as enzymes. RBApy extends the pool of functions to model complex kinetics, with functions such as the Arrhenius law to describe temperature-dependent enzyme catalytic rates. In addition, we made RBApy more customizable by allowing users to define new cellular processes and by avoiding any hard-coded biological information during model construction. The tool also provides a set of default cellular processes necessary to cell growth and survival for three categories of cells (prokaryotic, eukaryotic with and without chloroplasts), which can be refined during model construction.

#### Eukaryotic cell physiology

Unlike prokaryotic cells, eukaryotic cells are compartmentalized into organelles, resulting in multiple possible locations of molecular species and associated cellular processes. For instance, an enzyme may be present in multiple organelles while catalyzing the same reaction. Likewise, some cellular processes may occur across different organelles. For instance, in plant cells, protein translation takes place in the cytoplasm, mitochondria, and chloroplasts. RBApy handles multiple subcellular locations of molecules and macromolecules by creating localized instances and associates them with organelle-specific cellular processes. Differences in the biochemical composition of organelles (i.e. specific lipid composition of membranes, housekeeping proteins, free metabolites) can also be modeled. Finally, organism-specific coupling constraints, such as constraints imposing or coupling metabolic fluxes, enzyme abundances, or cellular organelle sizes, can be encoded or removed easily.

#### Stationary phase

Molecule abundances tend to decrease over time due to dilution by growth and molecule turnover, each molecule having its own lifespan. In RBApy, every macromolecule (such as proteins and RNAs) has a half-life and can be degraded by specific cellular processes (e.g. proteolysis for proteins), allowing its components (e.g. amino acids, ions, cofactors, nucleotides) to be recycled during the stationary phase (i.e. in the absence of dilution).

#### Context-specific RBA models

Genes, enzymes and metabolic reactions can be (permanently) removed during model construction using a curation file, which allows for building generic as well as context-specific RBA models. Constructing and curating a generic RBA model as a template can serve as a foundation for various context-specific model variants, thus strongly reducing curation effort.

### Model simulation and calibration

Simulating an RBA model with RBApy or RBAtools predicts, by default, the cell phenotype that maximizes the growth rate, i.e. the maximal possible growth rate, metabolic fluxes, abundances of molecular machines involved in metabolism and cell processes such as enzymes, ribosomes, etc., and the compartment sizes. Any model can be simulated in one of two ways: (i) by setting the compartment sizes to measured density values as in Bulović *et al.* 2019 or (ii) by optimizing these sizes during the simulation as in Goelzer *et al*. (2024).

#### Model complexity and tractability

One metabolic reaction can be catalyzed by several enzymes (called isoenzymes). This occurs in particular when one or several enzyme subunits are encoded by several paralogous genes, i.e. genes that originated from a duplication event and may retain similar functions. RBApy automatically creates models containing explicitly all possible isoenzymes for a given metabolic reaction. The model size could thus quickly increase, especially for polyploid organisms that have multiple isoenzymes per metabolic reaction. This might cause numerical instabilities due to singularities in the optimization problem during model simulation. To overcome this situation and ensure model simulation, RBApy also automatically creates an average RBA model pooling all isoenzymes catalyzing the same metabolic reaction into a single enzymatic complex, which has the average composition in amino acids, ions and cofactors of isoenzymes.

#### Model calibration

The RBApy calibration module has been updated to support the new features in RBApy, in particular the multiple localizations of macromolecules and the calibration of the average RBA model.

### FAIR principles

RBApy 3.0 generates a file structure for data, model (encoded in RBAml 2.0 format), and simulation results that follows the FAIR principles, implemented in the COMBINE archive specifications (https://co.mbine.org/). RBApy 3.0 adds metadata during model construction, such as authorship, generation or modification date, RBApy package version, etc., to improve RBA model traceability and reproducibility. Any existing RBA model can be automatically upgraded from RBAml 1.0 encoding format to RBAml 2.0. In particular, we tested the RBApy 3.0 simulation module by updating the plant cell model that runs in MATLAB (Goelzer *et al*. 2024) and by comparing the simulation results, and obtaining a perfect correspondence between predictions (see Supplementary Fig. 1).

## Modeling complex phenomena

### Temperature adaptation of *Arabidopsis thaliana*

Here, we demonstrate the new functionalities of RBApy by constructing a generic RBA cell model of the plant *A. thaliana* and using it to simulate temperature adaptation. Temperature adaptation is a particularly relevant use case because it simultaneously affects enzyme kinetics, protein stability, and cellular stress responses (Knapp and Huang 2022), making it a challenging benchmark for resource allocation models. We based our model on the GEM of *A. thaliana’*s leaf cell and the cell processes modeled in Goelzer *et al*. 2024 and completed both of them with paralogous genes that are not expressed in leaves. We then modeled the effect of temperature on cell growth by implementing temperature-dependent (i) turnover rates of proteins (Scheurwater *et al*. 2000); (ii) catalytic rates of enzymes and chaperones (Parent *et al*. 2010); (iii) chaperoning demand for protein folding; (iv) protein aggregation due to protein denaturation (cf. Fig. 1a); and (v) protein decay and proteolysis mediated by the 26S proteasome and serine proteases, the primary proteases active during the vegetative growth phase of *Arabidopsis* (Roberts *et al*. 2012). Values of temperature-dependent model parameters were extracted from the literature, except for the chaperoning demand and the protein aggregates after 28°C which were fitted manually in the absence of information (see Supplementary Material and Supplementary Figs 5 and 6). We simulated the RBA model for temperatures varying from 10°C to 45°C and compared the predicted growth rates to those reported in Parent and Tardieu (2012; see Fig. 1b), showing excellent agreement with the measurement data, especially at temperatures between 10 and 28°C, the temperature range for *Arabidopsis* growth. Protein allocation to different cell processes behaves as expected, with an increasing investment in gene expression processes (replication, transcription, translation) from 2% to 7% in agreement with increasing growth rate between 10 and 28°C, and with an increasing investment from 0.6% to 1.4% in folding and proteolysis between 28 and 36°C.

**Figure 1:**
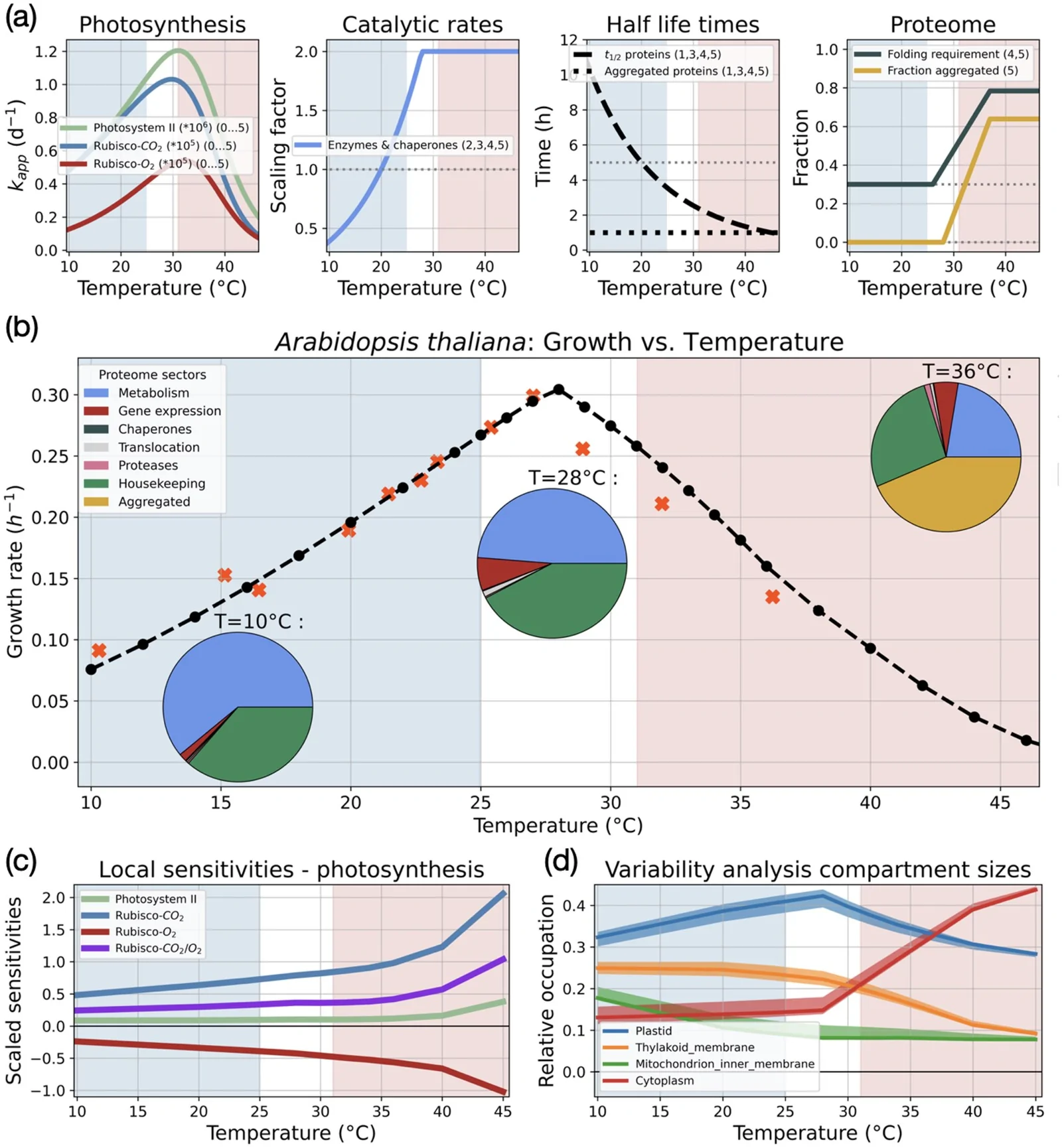
Modeling, simulating, and analyzing the temperature adaptation of *A. thaliana* with RBApy 3.0 and RBAtools 2.0. (a) Temperature-dependent parameters: catalytic rates of RuBisCO carboxylase (RuBisCO-CO_2_) and oxygenase (RuBisCO-O_2_), photosystem II modeled as in (Goelzer *et al*. 2024); scaling factor of other catalytic rates of molecular machines; protein half-life (aggregates or other proteins); and proteome fractions subject to aggregation (in cytoplasm) and chaperoning. (b) Predicted growth rate over screened temperatures and distribution of cellular protein across different proteome sectors at 10, 28, and 36°C. (growth rates, extracted from Parent and Tardieu 2012 are represented with a red cross for comparison). (c) Local sensitivity of fitness to RuBisCO carboxylase alone (blue), oxygenase alone (red), both simultaneously (purple), and PSII (light blue) catalytic rates on panel a. (d) Evolution of growth optimal compartment sizes and their feasible range (tolerance) at 95% suboptimal growth rates, obtained with RVA (resource variability analysis).

We further investigated the sensitivity of the predicted fitness (defined as the relative change in growth rate per relative change in parameter) for variations of RuBisCO and PSII catalytic rates, two enzymes involved in the photosynthetic apparatus and thus concentrating a large part of the leaf resources of *A. thaliana* (Fig. 1c). RuBisCO exhibits both carboxylase (i.e. carbon-fixing) and oxygenase (i.e. oxygen-fixing) activity, depending on whether CO_2_ or O_2_ binds to the catalytic site. Although fitness declines when only RuBisCO’s oxygenase activity is considered, increasing the catalytic activities of all RuBisCO-associated reactions has an overall positive effect, despite the inherent tradeoff between its oxygenase and carboxylase functions. Sensitivity to catalytic rates also increases with temperature. Since proteins aggregate at higher temperatures, the proteome resources available for metabolism decline. Consequently, metabolism must become more efficient at elevated temperatures to maintain function with fewer enzymes. This means that variations affecting metabolic efficiency, whether positive or negative as examined in our local sensitivity analysis, have a greater impact at higher temperatures, particularly for enzymes that constitute a significant fraction of the proteome, such as PSII and RuBisCO.

We finally determined the evolution of compartment sizes, modeled as the relative proteome partitioning across different organelles, and their tolerable ranges at 95% suboptimal growth rates by applying Resource Variability Analysis using RBAtools (Fig. 1d). The cytoplasm size is increasing with temperature, in agreement with the accumulation of cytoplasmic protein aggregates. The constant magnitude of compartment tolerance ranges indicates the absence of alternative cellular configurations (w.r.t. global proteome allocation across organelles) at suboptimal growth rate.

### Heat shock response of *Escherichia coli*

We extended the RBA model of wild-type *E. coli* which had originally been constructed and calibrated for growth on glucose minimal medium at 37°C (Bulović *et al.* 2019), to account for temperature-dependent parameters and cell processes (see Supplementary Fig. 2). We simulated the model for temperatures ranging from 20 to 46°C and compared the results to data reported in Chen *et al*. (2017). As for *Arabidopsis*, the predictions are in excellent agreement with the measurement data (see Supplementary Figs 3 and 4).

## Conclusion and perspectives

Here, we expanded the capabilities of the RBA toolkit (RBApy, RBAtool, RBAml) to incorporate unique characteristics of eukaryotic cells growing in complex environments such as varying temperature, light, or nutritional conditions. Using these tools, we constructed and simulated RBA models for both prokaryotic (*E. coli*) and eukaryotic (*A. thaliana*) organisms and predicted their growth and resource allocation behavior across a range of temperatures. The predictions proved to be highly accurate when benchmarked against published experimental datasets.

### Toward tissue-specific RBA models

Creating toward tissue-specific RBA from a generic RBA model necessitates the specialization of the biomass composition, the metabolic network, and molecular machines to the tissue or to the developmental stage. The workflow is thus similar to generating tissue-specific GEMs (tsGEM; Gopalakrishnan *et al*. 2023) and requires a gene expression dataset (transcriptomics or proteomics) collected on the tissue of interest. A tsGEM is first generated from a generic GEM by removing the genes and metabolic reactions that are not expressed in the tissue, by using for instance DEXOM (Rodríguez-Mier *et al*. 2021). Then the list of removed genes and reactions is extracted and is also removed from the generic RBA model (see Section ‘Model construction’). We thus anticipate that the generation of tissue-specific RBA models should be straightforward with RBApy.

### A step toward multicellular organism modeling

Altogether, the RBA toolkit can be used to model any prokaryotic or eukaryotic cell type, thus paving the way toward the modeling of multicellular organisms such as crops, livestock, or humans, for a wide range of medical, biotechnological or agricultural applications.

## Supplementary Material

vbag276_Supplementary_Data

## Data Availability

The data underlying this article are available at https://forge.inrae.fr/anne.goelzer/rbapy-3.0-use-case-models. RBApy and RBAtools are available via PyPI, code source and technical documentation are available at https://github.com/RBAgroup. Documentation for users is available online at https://rbagroup.github.io/RBApy/ and https://rbagroup.github.io/RBAtools/.
