# Supplementary material for: RBApy: extending resource allocation modeling to eukaryotes in complex environments
Source: Bioinform Adv. 2026 Sep 17;6(1):vbag276. doi: 10.1093/bioadv/vbag276 (PMC13626133; doi:10.1093/bioadv/vbag276)
Supplement: Supplementary file 1 [file vbag276_supplementary_data.pdf]

Supplementary material

RBApy: Extending resource allocation modeling to eukaryotes in complex environments

I. Supplementary material and methods

RBApy takes as input an annotated genome-scale metabolic model in SBML format (Hucka et al. 2004). In (Goelzer et al. 2024), we used an updated version of the AraCore model of (Arnold et al. 2014) for our metabolic model that originally modeled the leaf of *Arabidopsis thaliana.* In this paper, we updated the metabolic model again (i) to integrate the paralogues that are not expressed in leaves, (ii) to increase the interoperability of the metabolic model with the BIGG database (King et al. 2016), i.e. the database that compiles curated metabolic models for many organisms.

Addition of missing paralogue genes in the metabolic model.

We added missing paralogs to GPR rules by expanding gene sets within each subunit (OR relationships). For each added paralog, we checked whether it is involved in other reactions and whether its cellular localization is consistent with that of all considered reactions.
Paralogs were primarily retrieved from KEGG. For genes not found in KEGG (37 genes), we manually queried additional databases, including Ensembl via BioMart, TAIR, Phytozome, and UniProt. The objective was to select paralogs with high sequence similarity and shared biological functions. A total of 160 generalized reactions were identified among the 1,031 reactions, and 166 paralogs were added.

Interoperability with BIGG and KEGG databases. We mapped the original identifiers of cellular compartments and metabolic species of the metabolic model in (Goelzer et al. 2024) with the ones of the BIGG database (King et al. 2016). Cellular compartment identifiers were mapped manually, while metabolic species were mapped by web scraping of the metabolite name from the BIGG database. We automatically collected the most probable metabolite identifier and checked it manually. We also updated the KEGG reactions identifiers of the metabolic model with the current version of the database (March 2026; Kanehisa, M., et al. (2025)).

II. Validation of RBA core implementation

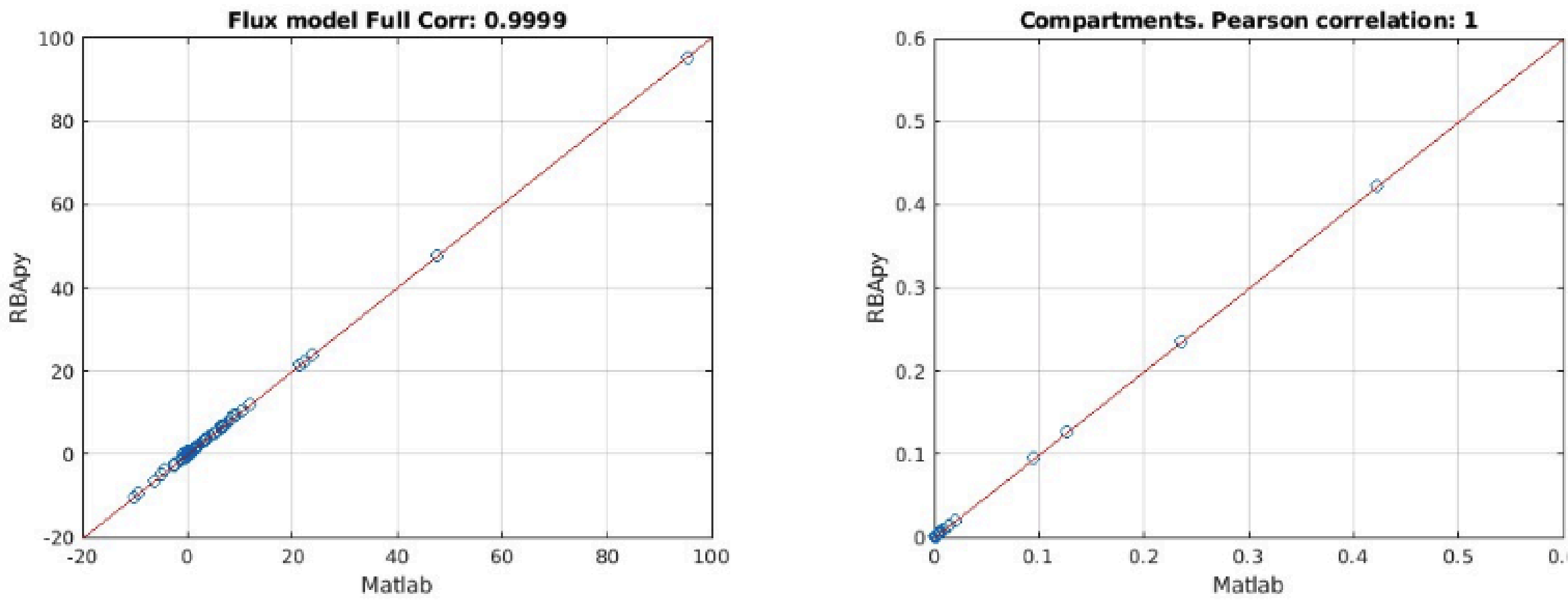

Supplementary figures 1. Comparison of Matlab and Python implementations for fluxes (in mM/gDW/day) (left) and compartments (fraction) (right).

III. Modeling temperature variation in RBA models

All scripts that are necessary to reproduce the figures and the results of this section are available at https://forge.inrae.fr/anne.goelzer/rbapy-3.0-use-case-models.

III.1 *Escherichia coli*

In this section, we present the RBA model of *Escherichia coli* that accounts for temperature variation, the different scenarios of model simulation and the results.

III.1.1 Model refinement

To simulate the cell response with respect to temperature variations, we changed the original model (Bulovic et al. 2019; https://github.com/RBAgroup/RBA-models/tree/master/Escherichia-coli-K12-WT) as follows:

- update the *E. coli* model with RBApy 3.0
- add two processes of protein degradation (Gottesman et al. 1996, Bittner et al. 2016, Bulovic 2023):
    - one mediated by the Lon protease that targets all cellular proteins
    - one mediated by ClpXP protease that targets only protein aggregates
- add one protein species with average composition (that will represent cytoplasmic protein aggregates during heat shock) whose concentration is set to 0 at 39°C and below, then is increasing linearly with temperature after 39°C (Govers et al. 2018)

III.1.2 Temperature-dependent parameters

All temperature-varying parameters and displayed in **Supplementary fig. 2** below:

- make the protein half-life to be a temperature-dependent parameter (choosing an Arrhenius law, centered at 37°C to a half-life of 15 hours, in the range of reported protein half-life (12 to 42 hours) by Maier et al. 2011). (See **left** panel in **Supplementary fig. 2**)
- make enzyme, ribosome and chaperone efficiencies to be temperature-dependent parameters as in (Bulovic, 2023). (See **center** panel in **Supplementary fig. 2**)

- make the input fraction of the proteome that necessitates chaperones to be folded to be a temperature-dependent parameter: the input fraction increases from baseline 10% (constant at T<=38°C) to 30% at 43°C (Tomoyasu, T., et al. 2001) and we decided to extrapolate this linear correlation until a fraction of 0.5 is reached. (See **right** panel in **Supplementary fig. 2**)
- define a temperature dependence for the fraction of protein aggregates among the proteome with a fraction of 0% until the onset of heat shock (T<=38°C) a maximum of 40% at 52°C. (See **right** panel in **Supplementary fig. 2**)
- define temperature-dependent efficiencies for the two proteases. In the absence of information, we assumed that the efficiencies of the Lon protease is equal to the temperature-dependent chaperone efficiency, and that the efficiency of the ClpXP protease degrading protein aggregates is 100-times less efficient than the others, because of the difficulty to degrade protein aggregates in cells.

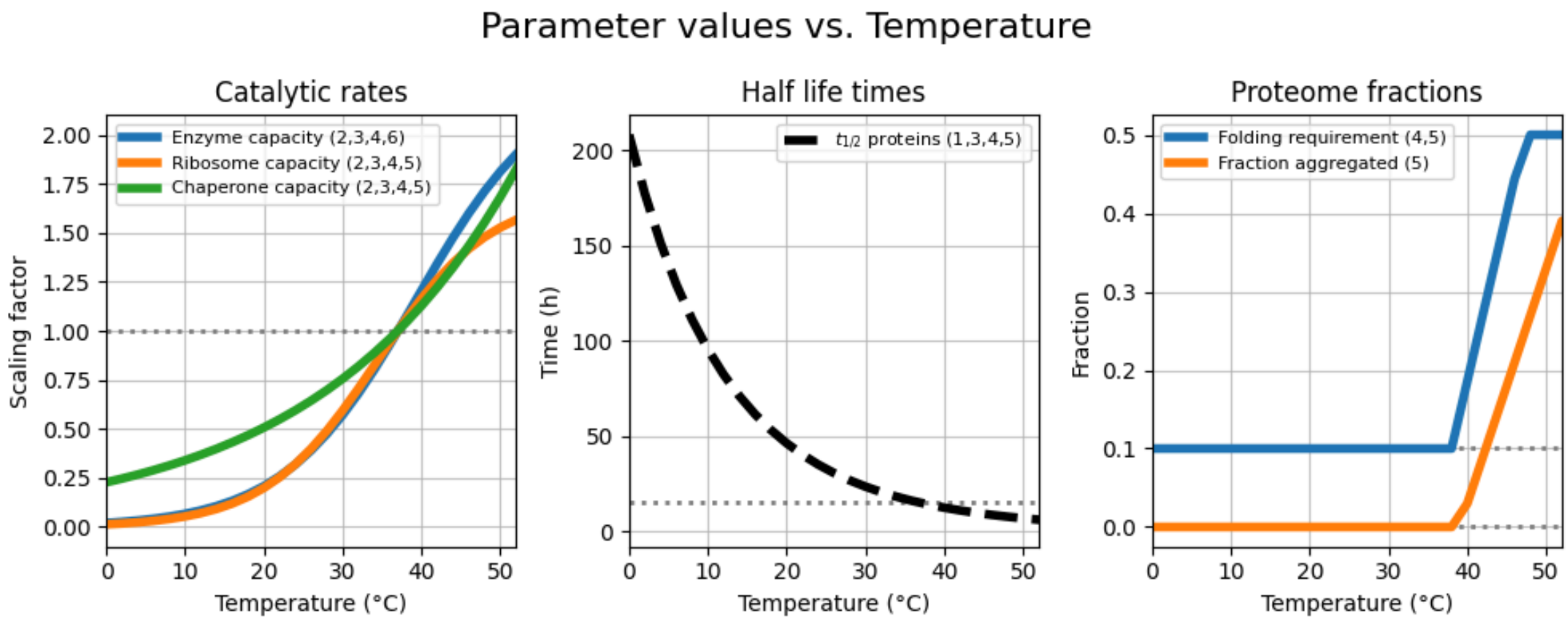

Supplementary figures 2. Temperature-dependent parameters in the RBA model of *Escherichia coli*: (left) enzyme, ribosome and chaperone efficiencies; (middle) protein half-life; (right) fraction of the proteome that is folded by chaperones (blue) or that aggregates (orange). Dashed lines represent constant values in the original temperature-independent scenarios for this parameter. Numbers in legend indicate in which tested scenarios the parameter is temperature-dependent.

Model calibration. The original temperature-independent RBA model was already calibrated on a glucose minimal medium (Bulovic et al. 2019). The choice of the temperature-dependent parameters (shape of curves and values) were directly extracted from literature (see above). Only one parameter, i.e. the fraction of protein aggregates for which we had no information, was fitted manually until we matched the (test-)data well (see red and green points in **Supplementary fig. 4**).

III.1.3 Simulations

To understand how each temperature effect contributes to the predicted cell phenotype, we introduced each effect individually into the RBA model and ran simulations across a temperature range of 20°C to 46°C (see **Supplementary fig. 3**):

- Scenario 0: original model with structural refinements no temperature effect (i.e. our reference condition). For constant parameter choices see dashed lines in **Supplementary fig. 2.**
- Scenario 1: add a centered Arrhenius law on each individual protein half-life (**Supplementary fig. 2 middle**)
- Scenario 2: add a logistic temperature-scaling on individual efficiencies of enzymes, transporters, ribosomes and exponential temperature scaling for chaperones (**Supplementary. fig. 2 left**), assuming that these efficiencies at 37°C coincide with the original calibrated RBA model (Bulovic et al. 2019)
- Scenario 3: scenario 1 + scenario 2
- Scenario 4: scenario 3 + add a temperature-dependent input fraction of proteins to folding (**Supplementary fig. 2 right**)
- Scenario 5: scenario 4 + a temperature-dependent fraction of the proteome that makes aggregates in the cytoplasm (**Supplementary fig. 2 right**)

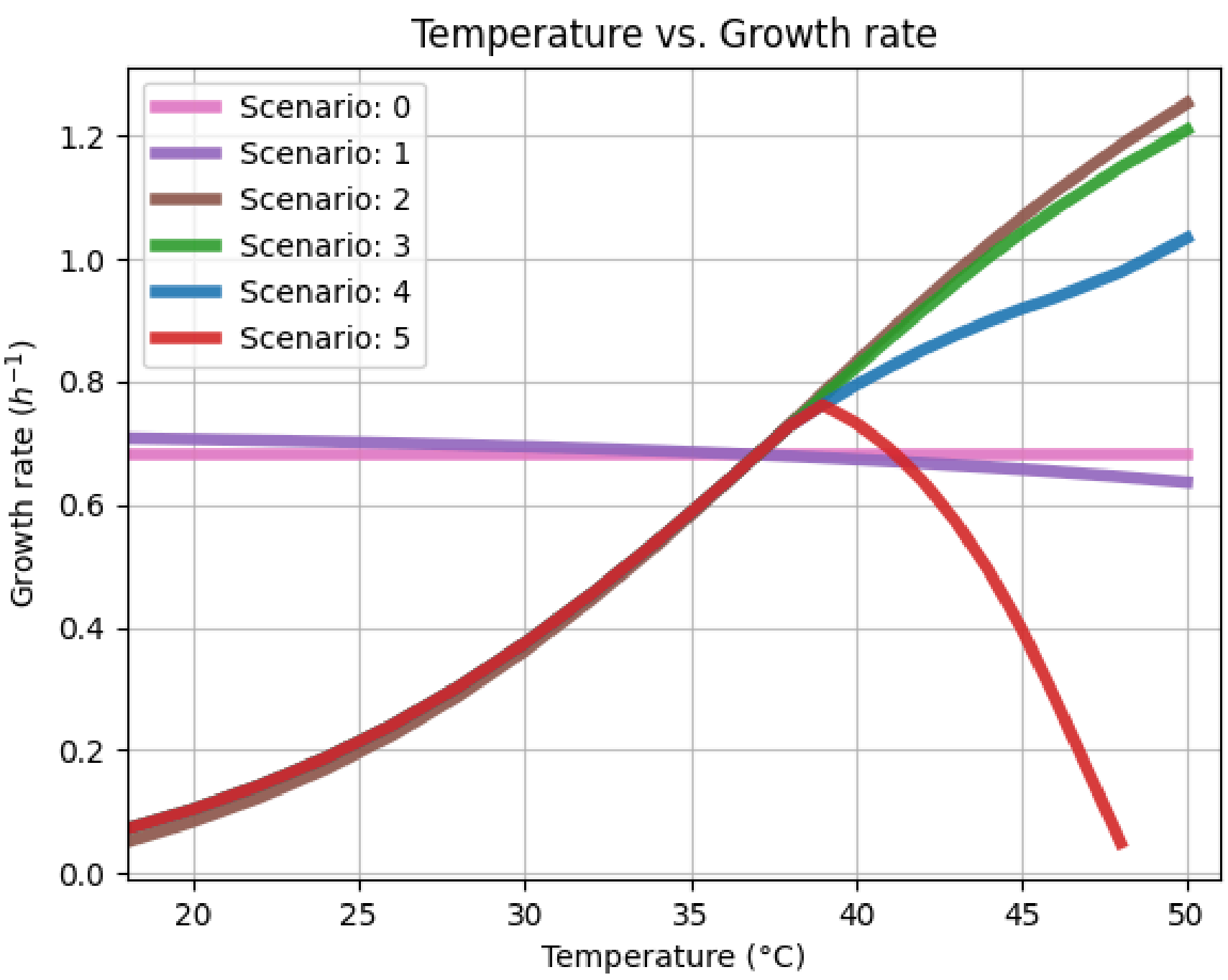

Supplementary figure 3. Predicted growth rate with respect to temperature for the five scenarios.

We finally compared the results of simulation for scenario 5 (i.e. the final RBA model accounting for all temperature effects) with data reported in (Chen et al. 2017). Predictions are in excellent agreement with the measurements (see **Supplementary figure 4**).

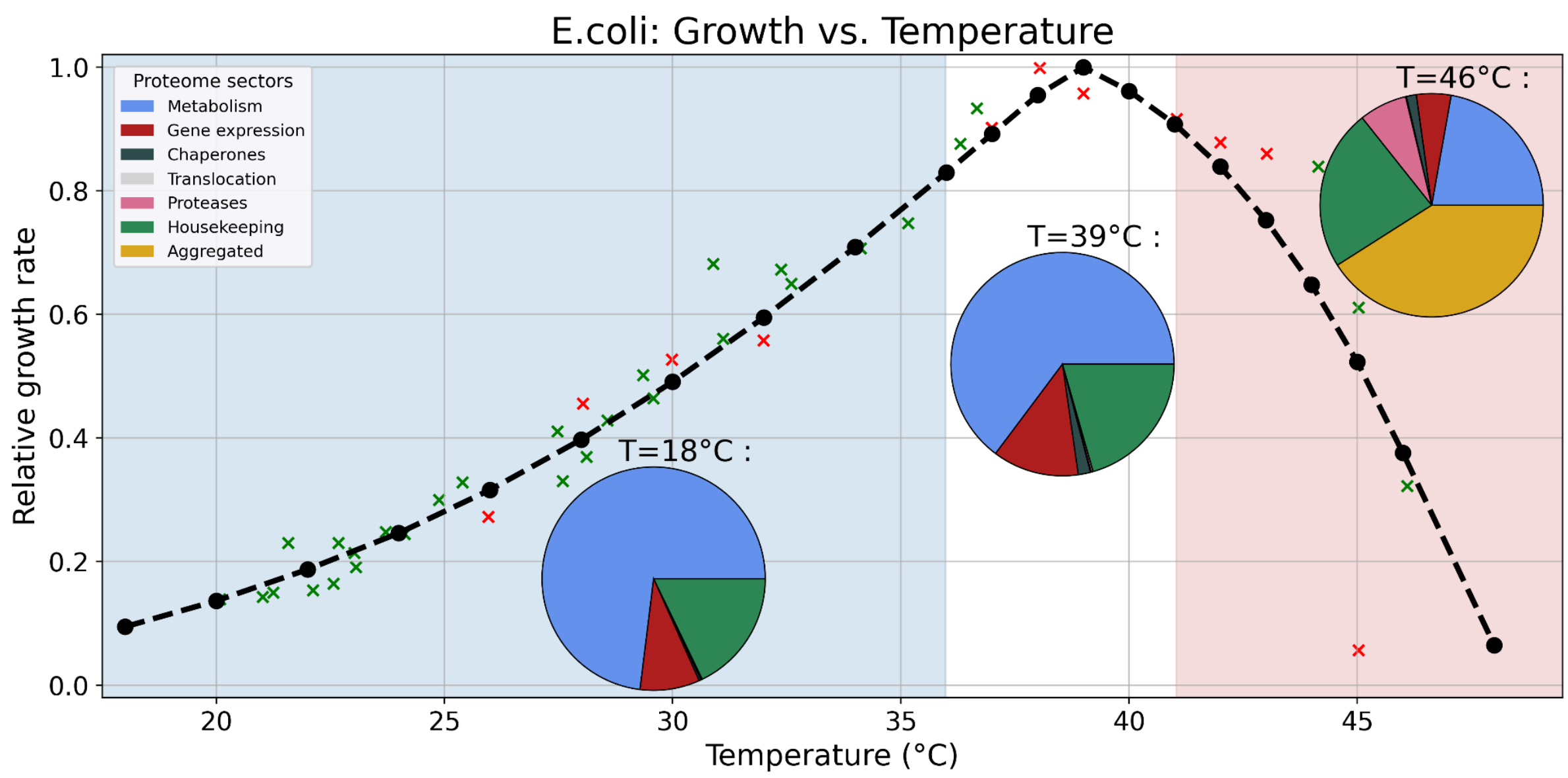

Supplementary figure 4. Predicted growth rate with respect to temperature using the RBA model accounting for all temperature effects. Reference data points in red and green were extracted from Chen et al. 2017. The red points correspond to experimental values by Chen et al. 2017 in M9+glucose medium and green points correspond to literature derived values in Chen et al. 2017, labelled BHI (brain-heart-infusion medium). Growth rates for the reference data and RBA predictions are given as fractions of the maximum growth rate, as in Chen et al. 2017. The maximal growth rate predicted by RBA is 0.76h^{-1} and is obtained at 39°C (see Supplementary figure 3).

III.2 *Arabidopsis thaliana*

In this section, we present the RBA model of *Arabidopsis thaliana* that accounts for temperature variation, the different scenarios of model simulation and the results.

III.2.1 Model refinement

To simulate the cell response with respect to temperature variations, we first built the generic RBA model using the same cellular processes as in (Goelzer et al. 2024), and which already incorporated temperature-dependent efficiencies for two enzymes, the photosystem II involved in photosynthesis, and the RuBisCO involved in carbon fixation, see **Supplementary. fig. 5 left**). Then we updated the model as follows:

- add the four major processes of protein degradation during vegetative growth (Roberts et al. 2012):
    - one mediated by the 26S proteasome located in the cytoplasm and that degrades any proteins located in the cytoplasm, the golgi, the endoplasmic reticulum, the peroxysome, the vacuole
    - one mediated by the ClpB1 (HSP101) heat shock protease located in the cytoplasm that degrades protein aggregates in the cytoplasm
    - one mediated by the ClpCP protease, located in the plastid and that degrades any proteins located in the plastid and the thylakoid
    - one mediated by the ClpXP protease, located in the mitochondrion and that degrades any proteins located in the mitochondrion

- add one protein aggregate (that will model protein aggregates during temperature elevation) located in the cytoplasm whose concentration is set to 0 at 25°C and below, then is increasing linearly with temperature after 28°C.

III.2.2 Temperature dependent parameters

- make the protein half-life to be a temperature-dependent parameter (using an Arrhenius law, centered at 20°C at a protein half-life of 5 days, in the range of reported total protein half-life (4 to 6 days) in Scheurwater, et al. 2000) (see **Supplementary. fig. 5 2nd panel from right**).
- make enzyme and chaperone efficiencies temperature-dependent parameters; doubling every 10°C between 10°C and 28°C, anchored at 20°C (Parent et al. 2010) (see **Supplementary. fig. 5 2nd panel from left**).
- make the chaperoning demand, i.e. the input fraction of chaperones (the fraction of proteins necessitating chaperones to be folded) to be temperature-dependent parameters: the input fraction increases from 30% to 80% between 27°C and 37°C (see **Supplementary. fig. 5 right**).
- define a temperature dependence for the protein aggregate fraction of 0% until the onset of heat shock (T<=28°C) and a linear increase until 60% of the cytosolic proteome at 37°C. (See **right** panel in **Supplementary fig. 5 right**)
- define temperature-dependent efficiencies for the four proteases. In the absence of information, we assumed that the efficiencies of the 26S proteasome, ClpXP and ClpCP proteases are equal to the temperature-dependent chaperone efficiency, and that the efficiency of the CLPB1 protease degrading protein aggregates is 100-times less efficient than the others, because of the difficulty to degrade protein aggregates in cells.

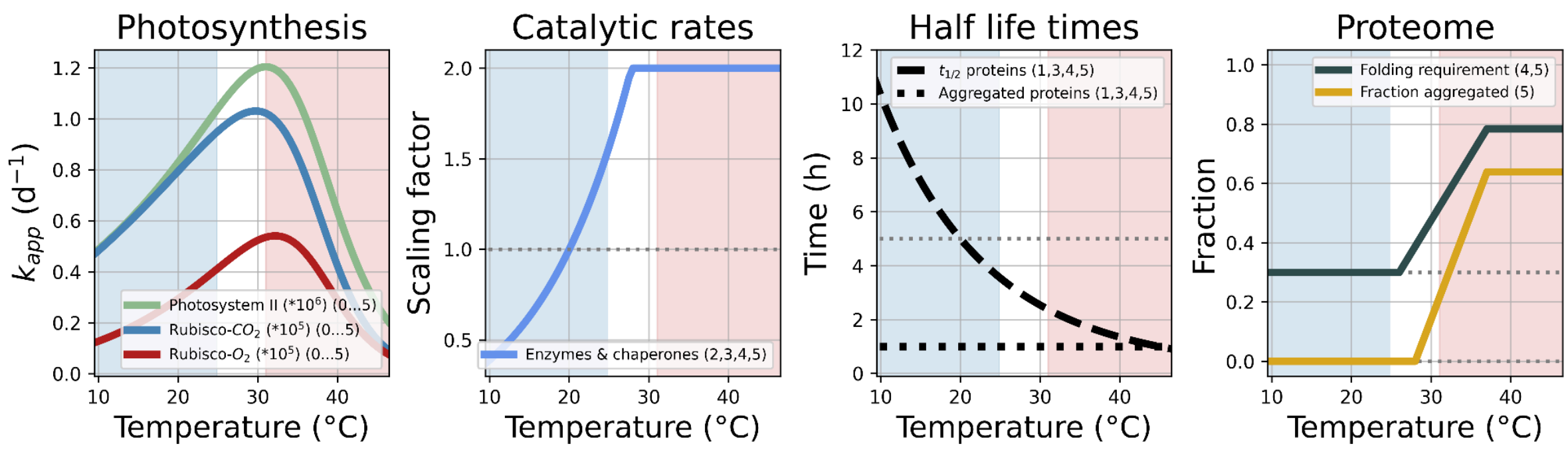

Supplementary figures 5. Temperature-dependent parameters in the RBA model of *Arabidopsis thaliana*: (1) Catalytic rates of RuBisCO carboxylase (RuBisCO-CO_2) and oxygenase (RuBisCO-O_2), of Photosystem II modeled as in (Goelzer et al. 2024); (2) Scaling factor of (other) catalytic rates of molecular machines; (3) Protein half-life (aggregates and other proteins); and (4) Proteome fractions subject to aggregation (in cytoplasm) and chaperoning. Numbers in legend indicate in which tested scenarios the parameter is temperature-dependent.

Model calibration. The original RBA model was already calibrated using realistic parameters (Goelzer et al. 2024), which remained identical in the new

temperature-dependent RBA model. The choice of the temperature-dependent parameters (shape of curves and values) were directly extracted from literature (see above). We capped the temperature dependence of enzyme and transporter efficiencies after 28°C, the optimal temperature for Arabidopsis. Only two parameters, i.e. the fraction of protein aggregates and the chaperoning demand for which we had no information, were fitted manually until we matched the (test-)data well (see reference data points in fig 1-b in the main text).

III.2.3 Simulations

To understand how each temperature effect contributes to the predicted cell phenotype, we introduced each effect individually into the RBA model and ran simulations across a temperature range of 10°C to 45°C (see Supplementary figure 6):

- Scenario 0: no temperature effect except Photosystem II and RuBisCO (i.e. our reference condition) as in (Goelzer et al. 2024)
- Scenario 1: add a centered Arrhenius law on each individual protein half-life, assuming a value of 5 days at 20°C (**Supplementary. fig. 5 2nd panel from right**)
- Scenario 2: add a logistic temperature-scaling on individual efficiencies of enzymes, and chaperones (**Supplementary. fig. 5 2nd panel from left**), assuming that these efficiencies at 20°C coincide with the original RBA model (Goelzer et al. 2024)
- Scenario 3: scenario 1 + scenario 2
- Scenario 4: scenario 3 + add a temperature-dependent input fraction of proteins to folding in the cytoplasm (**Supplementary. fig. 5 right**)
- Scenario 5: scenario 4 + a temperature-dependent fraction of the proteome that makes aggregates in the cytoplasm (**Supplementary. fig. 5 right**)

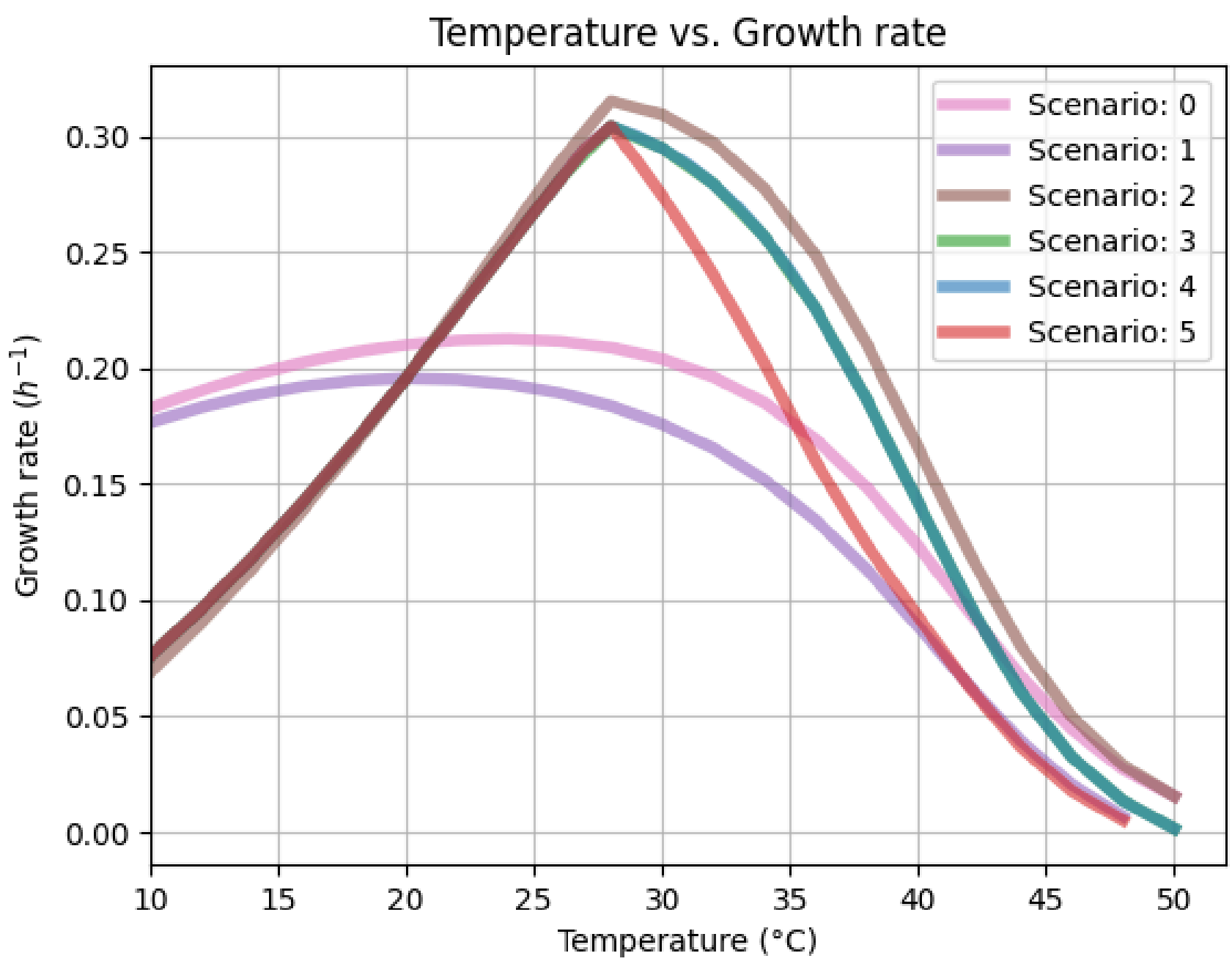

Supplementary figure 6. Predicted relative growth rate with respect to temperature for the five scenarios.

We finally compared the predicted relative growth rate of the last scenario (i.e. the final RBA model accounting for all temperature effects) with the one reported in (Parent and Tardieu 2012) (see Figure 1b of the paper).

IV. Declaration of AI-assisted technologies in the manuscript preparation process.

During the preparation of this work the authors used Mistral AI in order to improve the clarity and readability of the manuscript. After using this tool/service, the authors reviewed and edited the content as needed and took full responsibility for the content of the published article.

V. References

Arnold, A., and Nikoloski, Z. (2014). Bottom-up metabolic reconstruction of Arabidopsis and its application to determining the metabolic costs of enzyme production. *Plant physiology*, *165*(3), 1380-1391.

Bittner, L. M., et al. (2016). Mini review: ATP-dependent proteases in bacteria. *Biopolymers*, *105*(8), 505-517.

Bulovic, Ana (2023). *Regulatory systems of relevance for bioproduction*. Diss. humboldt university, Berlin, Germany.

Bulović, A., *et al*. (2019). Automated generation of bacterial resource allocation models. *Metab. Eng. 55*, 12-22.

Chen, K., et al. (2017). Thermosensitivity of growth is determined by chaperone-mediated proteome reallocation. Proceedings of the National Academy of Sciences, 114(43), 11548-11553.

Goelzer, A., et al. (2024). Resource allocation modeling for autonomous prediction of plants cell phenotypes. Metab. Eng., 83, 86-101.

Gottesman, S. (1996). Proteases and their targets in Escherichia coli. Annual review of genetics, 30(1), 465-506.

Govers, S. et al. (2018). Protein aggregates encode epigenetic memory of stressful encounters in individual Escherichia coli cells. *PLoS biology*, *16*(8), e2003853.

Hucka, M. *et al.*The systems biology markup language (SBML): a medium for representation and exchange of biochemical network models. Bioinformatics, 19 (4) (2003), pp. 524-531

Kanehisa, M., et al. (2025). KEGG: biological systems database as a model of the real world. *Nucleic acids research*, *53*(D1), D672-D677.

King, Z. A., et al. (2016). BiGG Models: A platform for integrating, standardizing and sharing genome-scale models. *Nucleic acids research*, *44*(D1), D515-D522.

Maier, T., et al. (2011). Quantification of mRNA and protein and integration with protein turnover in a bacterium. *Molecular systems biology*, *7*(1), MSB201138.

Parent, B., et al. (2010). Modelling temperature-compensated physiological rates, based on the co-ordination of responses to temperature of developmental processes. *Journal of experimental botany*, *61*(8), 2057–2069.

Parent, B., and Tardieu, F. (2012). Temperature responses of developmental processes have not been affected by breeding in different ecological areas for 17 crop species. New Phytologist, 194(3), 760-774.
Roberts, I, et al. (2012). Senescence‐associated proteases in plants. Physiol. Plant., 145(1), 130-139.
Scheurwater, I., et al. (2000). Respiratory costs and rate of protein turnover in the roots of a fast‐growing (Dactylis glomerata L.) and a slow‐growing (Festuca ovina L.) grass species. *Journal of Experimental Botany*, *51*(347), 1089-1097.
Tomoyasu, T., et al. (2001). Genetic dissection of the roles of chaperones and proteases in protein folding and degradation in the Escherichia coli cytosol. *Molecular microbiology*, *40*(2), 397-413.
